# Dysregulation of Aurora Kinases and *AURKAIP1* Promoter Methylation as Potential Peripheral Diagnostic Biomarkers in Acute Myeloid Leukemia

**DOI:** 10.3390/cimb48040378

**Published:** 2026-04-05

**Authors:** Zubeyde Yalniz Kayim, Seref Bugra Tuncer, Betul Celik Demirbas, Ugur Gezer, Ozge Sukruoglu Erdogan, Seda Kilic Erciyas, Nejat Dalay, Akif Selim Yavuz, Vildan Yasasever

**Affiliations:** 1 Department of Cancer Genetics, Oncology Institute, Istanbul University, Istanbul 34093, Türkiye; seref.tuncer@istanbul.edu.tr (S.B.T.); betul.celik@istanbul.edu.tr (B.C.D.); ozge.erdogan@istanbul.edu.tr (O.S.E.); seda.kilic@istanbul.edu.tr (S.K.E.); 2 Department of Basic Oncology, Oncology Institute, Istanbul University, Istanbul 34093, Türkiye; ugurd@istanbul.edu.tr (U.G.); ndalay@yahoo.com (N.D.); yasasever@yahoo.com (V.Y.); 3 Faculty of Medicine, Department of Internal Medicine, Division of Hematology, Istanbul University, Istanbul 34093, Türkiye; yavuzas@istanbul.edu.tr

**Keywords:** acute myeloid leukemia, aurora kinases, gene expression, DNA methylation

## Abstract

Acute myeloid leukemia (AML) is an aggressive hematologic malignancy characterized by impaired differentiation and accumulation of immature myeloid cells. Aurora kinases and their regulatory genes play key roles in mitotic progression and may contribute to leukemogenesis. This study aimed to evaluate the expression and promoter methylation status of *AURKA*, *AURKB*, and *AURKC* and their regulatory genes, *AURKAIP1*, *E2F1*, and *E2F4* in AML. Peripheral blood samples from 83 AML patients and 28 age- and sex-matched healthy controls were analyzed using MIQE-compliant RT-qPCR for gene expression and MSRE-qPCR for promoter methylation. Diagnostic performance was assessed using receiver operating characteristic (ROC) curve analysis. Expression levels of *AURKA*, *AURKB*, *AURKC*, and *E2F1* were significantly increased in AML patients (*p* < 0.001), whereas *AURKAIP1* expression was significantly reduced (*p* = 0.001), and *E2F4* showed no significant difference. Promoter methylation analysis revealed significantly increased *AURKAIP1* methylation in AML (*p* < 0.001), decreased *E2F4* methylation (*p* = 0.023), and no significant change in *E2F1*. ROC analysis demonstrated strong diagnostic performance, with *AURKB* showing the highest accuracy (AUC = 0.95), while a combined biomarker panel achieved an AUC of 0.96. Aurora kinase–related genes are dysregulated in AML and may serve as preliminary peripheral biomarker candidates. However, further validation in independent cohorts and more refined cellular models is required before clinical application.

## 1. Introduction

Acute myeloid leukemia (AML) is an aggressive hematologic malignancy characterized by the clonal expansion of myeloid progenitor cells that fail to differentiate into mature blood cells [1]. The disease arises from a combination of genetic mutations and epigenetic alterations that disrupt normal hematopoiesis, leading to the accumulation of immature blasts in the bone marrow and peripheral blood [2]. Despite advances in targeted therapies and improved diagnostic stratification, AML continues to be associated with high relapse rates and poor overall survival, particularly among older patients [3,4].

In eukaryotic cells, proliferation is tightly regulated by cell-cycle checkpoints, particularly during the G1/S transition and mitosis [5]. Disruption of mitotic regulators can lead to chromosomal instability, a hallmark of leukemia [6]. Aurora kinases are a family of serine/threonine kinases essential for mitotic processes such as centrosome maturation, chromosome alignment, spindle assembly, and cytokinesis [7]. The Aurora kinase family consists of three members: *AURKA*, *AURKB*, and *AURKC*. *AURKA* primarily regulates centrosome function and spindle assembly during early mitosis, whereas *AURKB* plays a central role in chromosome condensation, kinetochore–microtubule attachment, and cytokinesis as part of the chromosomal passenger complex. *AURKC* is mainly expressed in germ cells and is involved in meiotic division [7]. Dysregulation of Aurora kinases has been widely reported in several malignancies, including AML, where they promote abnormal cell division, genomic instability, and resistance to therapy [8]. *AURKB*, in particular, is critical for accurate chromosomal alignment during mitosis, and its abnormal activity has been associated with increased cellular proliferation and resistance to therapy in AML patients [9]. Experimental studies have shown that Aurora kinase inhibition induces apoptosis in leukemic cells and enhances the efficacy of conventional chemotherapeutic agents, supporting its potential as a therapeutic target [10]. For example, the pan-Aurora kinase inhibitor MK-0457 has been shown to promote leukemic cell death and enhance the efficacy of standard therapies, supporting the therapeutic potential of Aurora kinase targeting in AML [11]. Moreover, inhibition of *AURKB* may help overcome treatment resistance mechanisms in AML, potentially improving clinical outcomes [12]. Collectively, these findings highlight Aurora kinases as promising molecular targets for AML therapy and underscore the need for further clinical investigation.

Beyond the Aurora kinase genes themselves, several regulatory proteins modulate their activity and downstream signaling [7]. *AURKAIP1* is a mitochondrial-associated protein that interacts with *AURKA* and is thought to regulate its stability and function [13]. It has also been implicated in apoptosis and mitochondrial dynamics, suggesting a potential role in cellular stress responses [14]. While promoter methylation has been associated with transcriptional regulation in various malignancies, the extent to which this relationship applies to *AURKAIP1* in AML remains unclear, particularly given the lack of consistent correlation between methylation status and gene expression. Therefore, further investigation is required to clarify the functional relationship between *AURKAIP1* methylation and gene expression in AML.

In addition, *E2F1* [15] and *E2F4* [16] are key transcription factors involved in cell-cycle regulation. *E2F1* promotes the transition from G1 to S phase by activating genes required for DNA replication while also playing a role in apoptosis [17]. *E2F4*, in contrast, is generally associated with transcriptional repression and cell-cycle exit [16]. Aberrant activation of *E2F1* has been widely linked to uncontrolled proliferation and cancer development, including leukemia [18]. However, the coordinated expression patterns and epigenetic regulation of these genes in AML remain poorly understood.

Promoter methylation is an important epigenetic mechanism capable of silencing tumor suppressor genes or enhancing oncogenic pathways [19]. In AML, abnormal methylation profiles have been associated with disease subtype, prognosis, and therapeutic response [20]. Nevertheless, the methylation status of genes regulating Aurora kinase activity has been insufficiently explored. Therefore, the present study aimed to evaluate the expression levels of *AURKA*, *AURKB*, and *AURKC* and the promoter methylation status of these genes together with their regulatory partners *AURKAIP1*, *E2F1*, and *E2F4* in AML patients compared with healthy controls, to clarify their roles in AML pathogenesis and assess their potential as diagnostic and prognostic biomarkers. This integrated approach may provide novel insights into the interplay between mitotic regulation and epigenetic alterations in AML.

## 2. Materials and Methods

### 2.1. Sample Collection

In this study, the expression levels of *AURKA*, *AURKB*, and *AURKC* and the promoter methylation status of their regulatory genes, *AURKAIP1*, *E2F1*, and *E2F4*, were evaluated in patients with AML and compared with healthy controls. A total of 83 AML patients diagnosed with AML between 2008 and 2013 at the Department of Hematology, Istanbul University Faculty of Medicine, were included (mean age: 44.98 ± 18.23 years). Among them, 83 peripheral blood samples were analyzed. The control group consisted of peripheral blood samples from 28 healthy individuals without a history of malignancy, matched for age and sex (mean age: 46.21 ± 9.98 years). Laboratory analyses were conducted at the Department of Basic Oncology, Oncology Institute, Istanbul University. Written informed consent was obtained from all participants before their enrollment in the study. The study protocol was approved by the Istanbul University Clinical Research Ethics Committee (approval date: 4 November 2009; approval no: 04), and all procedures were performed in accordance with the Declaration of Helsinki [21].

#### 2.1.1. Real-Time Quantitative PCR Analysis (MIQE-Compliant)

Total RNA was isolated from peripheral blood samples using the InviTrap Spin Universal RNA Mini Kit (STRATEC Molecular GmbH, Berlin, Germany). RNA integrity was confirmed by agarose gel electrophoresis, and RNA concentration and purity were measured spectrophotometrically using a NanoDrop 1000 (Thermo Fisher Scientific, Wilmington, DE, USA). Complementary DNA (cDNA) was synthesized from 100 ng of total RNA using the RevertAid First Strand cDNA Synthesis Kit (Thermo Fisher Scientific, Vilnius, Lithuania) according to the manufacturer’s instructions.

Real-time quantitative PCR (RT-qPCR) was performed on an ABI 7500 Real-Time PCR System (Applied Biosystems, Foster City, CA, USA) using TaqMan MGB hydrolysis probes. Each reaction was carried out in a final volume of 20 µL containing 10 µL TaqMan Universal PCR Master Mix (2×), 1 µL TaqMan Gene Expression Assay mix, 2.5 µL cDNA template, and nuclease-free water. All reactions were performed in duplicate, and no-template controls were included to exclude contamination.

The thermal cycling conditions were as follows: initial denaturation at 95 °C for 10 min, followed by 40 cycles of denaturation at 95 °C for 15 s and annealing/extension at 60 °C for 60 s. Fluorescence data were collected at each amplification cycle. PCR efficiency was confirmed to be within acceptable MIQE limits (90–110%) using standard curve analysis.

Relative gene expression levels were calculated using the 2^−ΔΔCt^ method, where ΔCt = Ct_target − Ct_reference. The *ZNF207* gene was used as an endogenous reference due to its stable expression across samples [22]. Primers used for gene expression analysis are listed in Table 1.

#### 2.1.2. DNA Isolation

Peripheral blood samples were subjected to density gradient centrifugation to isolate peripheral blood mononuclear cells (PBMCs), including lymphocytes, monocytes, and circulating leukemic blasts. Genomic DNA was then extracted using a commercially available kit (AccuPrep Genomic DNA Extraction Kit, Bioneer Corporation, Daejeon, South Korea) according to the manufacturer’s instructions. The isolated DNA samples were further purified using the DNA Clean & Concentrator Kit (Zymo Research, Irvine, CA, USA). DNA concentration and purity were assessed spectrophotometrically using a NanoDrop 1000 system (Thermo Fisher Scientific, Wilmington, DE, USA). DNA samples were diluted to a final concentration of 4 ng/µL and stored at −80 °C until further analysis. PBMCs comprise a heterogeneous population of lymphocytes, monocytes, and circulating leukemic blasts. In patients with AML, peripheral blood may contain varying proportions of blast cells at diagnosis; therefore, PBMC-derived nucleic acids are commonly used to assess disease-associated molecular alterations [23]. All samples were processed using a standardized protocol to minimize technical variability across the cohort.

#### 2.1.3. MSRE-qPCR–Based DNA Methylation Analysis

DNA methylation analysis was performed using an MSRE-qPCR approach with the OneStep qMethyl™ Kit (D5310, Zymo Research, Irvine, CA, USA). This method enables direct quantification of promoter methylation without bisulfite conversion and is well-suited for clinical samples with limited DNA input.

Genomic DNA was digested using methylation-sensitive restriction enzymes recognizing CpG-containing sequences, including AccII, HpaII, and HpyCH4IV. Digested DNA was then subjected to real-time PCR amplification on an Applied Biosystems Real-Time PCR System using gene-specific primers targeting the promoter regions of *AURKAIP1*, *E2F1*, and *E2F4*.

Each 20 µL PCR reaction contained 10 µL 2× reaction mix, 1 µL forward primer (10 µM), 1 µL reverse primer (10 µM), 5 µL DNA template (4 ng), and 3 µL nuclease-free water. Both test (enzyme-digested) and reference (undigested) reactions were prepared for each sample. Thermal cycling conditions were as follows: enzyme digestion at 37 °C for 2 h, initial denaturation at 95 °C for 10 min, followed by 40 cycles of denaturation at 95 °C for 30 s, annealing at 54 °C for 60 s, extension at 72 °C for 60 s, and a final extension at 72 °C for 7 min. Primer specificity and amplification efficiency for methylation assays were evaluated during assay optimization. Only primer pairs yielding specific amplification and consistent Ct values across control samples were included in the analysis. Internal controls provided by the kit were used to confirm assay performance and digestion efficiency.

Methylation levels were calculated according to the kit protocol using the formula:

% Methylation = 100 × 2^−ΔCt^,

ΔCt = Ct-test − Ct-reference

Samples with methylation values < 6% were considered methylation-negative, whereas values ≥ 6% were considered methylation-positive. The use of a binary classification threshold (≥6% vs. <6%) is based on the manufacturer’s validated criteria, which are designed to distinguish biologically meaningful methylation from background signal. This threshold has been widely applied in MSRE-qPCR–based studies and allows consistent comparison across samples. In the context of AML, such classification enables the identification of samples with detectable promoter methylation, which may reflect epigenetic alterations associated with disease status. Fully methylated and unmethylated DNA standards supplied in the kit, along with *MGMT* gene primers, were used as internal controls. Promoter regions and CpG islands of the *AURKAIP1*, *E2F1*, and *E2F4* genes were identified in silico, and primers were designed using Primer3 software (https://primer3.ut.ee/, accessed on 15 June 2014) to include restriction enzyme recognition sites. The selected promoter regions were chosen based on in silico analysis of CpG islands and proximity to transcription start sites (TSS). Primers were specifically designed to target CpG-rich regions located within or near promoter-associated CpG islands, which are known to play a critical role in transcriptional regulation. Therefore, methylation changes detected in these regions are more likely to have functional relevance for gene expression. Primer sequences that used for methylation analysis are provided in Table 2. The 6% threshold was determined according to the manufacturer’s recommendations in the OneStep qMethyl™ Kit manual (D5310, Zymo Research Corp., Irvine, CA, USA), which defines it as the standard cutoff for methylation analysis. Accordingly, all methylation measurements and sample classifications in this study were conducted in compliance with these validated criteria [24,25].

### 2.2. Statistical Analysis

Statistical analyses were performed using SPSS version 22.0 (IBM Corp., Armonk, NY, USA) and MedCalc Statistical Software version 23.2.0 (MedCalc Software Ltd., Ostend, Belgium). Continuous variables were expressed as median (interquartile range, IQR) or mean ± standard deviation (SD), as appropriate. The normality of data distribution was assessed using the Kolmogorov–Smirnov test. Comparisons between AML patients and healthy controls were performed using the Mann–Whitney U test for non-normally distributed variables and the independent samples *t*-test for normally distributed variables. Categorical variables were compared using the chi-square test or Fisher’s exact test, as appropriate. Receiver operating characteristic (ROC) curve analysis was performed to evaluate the diagnostic performance of individual genes and combined biomarker panels. The area under the curve (AUC), sensitivity, specificity, and 95% confidence intervals (CI) were calculated. Univariate logistic regression analysis was conducted to assess the association between gene expression levels and AML status. Results were presented as odds ratios (ORs) with 95% confidence intervals (CIs). Given the number of comparisons performed, false discovery rate (FDR) correction was applied using the Benjamini–Hochberg method to control for multiple testing, and adjusted *p*-values are provided in the Supplementary Material. A two-sided *p*-value < 0.05 was considered statistically significant.

## 3. Results

### 3.1. Patient Characteristics

A total of 83 patients with AML diagnosed between 2008 and 2013 at the Department of Hematology, Istanbul University Faculty of Medicine, were included (mean age 44.98 ± 18.23 years). The cohort consisted of 83 peripheral blood samples. The control group included 28 healthy individuals matched for age and sex (mean age 46.21 ± 9.98 years). The demographic and clinical characteristics of the study population are summarized in Table 3.

### 3.2. Expression Levels of Target Genes

**Figure 1:**
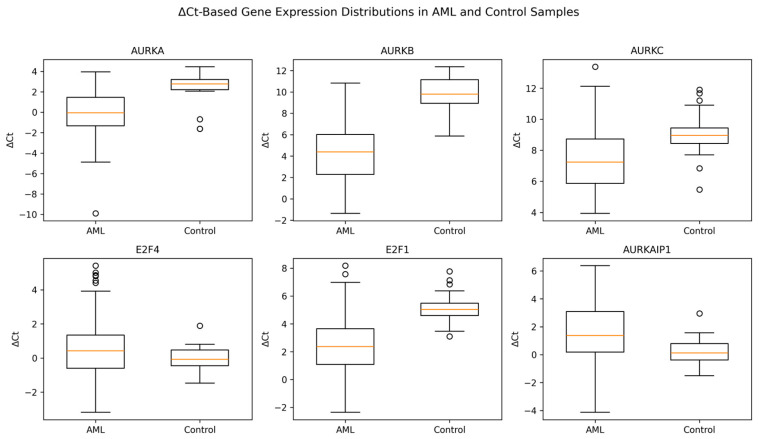
Distribution of gene expression levels in AML patients and healthy controls. Box plots represent ΔCt values for *AURKA*, *AURKB*, *AURKC*, *E2F4*, *E2F1*, and *AURKAIP1*. Lower ΔCt values indicate higher relative gene expression. The central line represents the median, boxes indicate the interquartile range (IQR), whiskers denote the range, and circles represent outliers. AML samples showed lower ΔCt values for *AURKA*, *AURKB*, *AURKC*, *and E2F1*, consistent with increased expression, whereas *AURKAIP1* showed higher ΔCt values in AML, indicating reduced expression. *E2F4* showed no significant difference between groups.

Expression levels of *AURKA*, *AURKB*, *AURKC*, *E2F1*, *E2F4*, and *AURKAIP1* were analyzed by RT-qPCR in AML patients and healthy controls using continuous 2^−ΔΔCt^ values. The distribution of gene expression levels between groups is illustrated in Figure 1 using Δ^Ct^-based box plots. *AURKA*, *AURKB*, *AURKC*, and *E2F1* expression levels were significantly higher in AML patients compared with controls (*p* < 0.001 for all), as reflected by lower ΔCt values in the AML group. In contrast, *AURKAIP1* expression was significantly reduced in AML patients (*p* = 0.001), corresponding to higher ΔCt values. No significant difference was observed for *E2F4* expression between AML and control groups (*p* = 0.16), with partially overlapping distributions. Overall, ΔCt-based box plot analysis demonstrated clear shifts in expression distributions between AML and control samples for Aurora kinase genes and *E2F1*, while confirming a gene-specific dysregulation pattern rather than a uniform change across all markers. After false discovery rate (FDR) correction using the Benjamini–Hochberg method, all previously significant associations remained statistically significant, further supporting the robustness of the results (Supplementary Table S1).

#### 3.2.1. DNA Methylation Analyses

**Figure 2:**
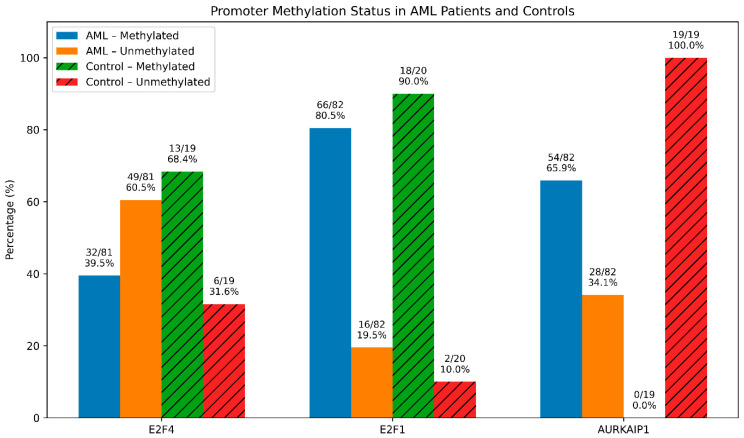
Promoter methylation status of *E2F4*, *E2F1*, and *AURKAIP1* in AML patients and healthy controls. (Bars represent the percentage of methylated and unmethylated samples in each group. Values above bars indicate sample numbers and percentages. Samples that failed amplification or digestion quality control were excluded from the relevant analyses).

***E2F4* Promoter Methylation:** Promoter methylation of *E2F4* was detected in 39.5% of AML patients (32/81) and 68.4% of controls (13/19). Methylation levels were significantly lower in AML patients compared with controls (*p* = 0.023; Figure 2). No association was observed between *E2F4* methylation and gene expression levels. ***E2F1* Promoter Methylation:** *E2F1* promoter methylation was detected in 80.5% of AML patients (66/82) and 90% of controls (18/20). The difference between groups was not statistically significant (Figure 2), and no correlation was found between methylation status and gene expression. ***AURKAIP1* Promoter Methylation:** Promoter methylation of *AURKAIP1* was observed in 65.9% of AML patients (54/82) but in none of the healthy controls. This difference was highly significant (*p* < 0.001; Figure 2). However, no association was detected between *AURKAIP1* methylation status and its gene expression levels or *AURKA* expression. The lack of correlation between methylation and gene expression suggests that additional regulatory mechanisms beyond promoter methylation may be involved.

Subgroup analyses showed no significant associations between *E2F4*, *E2F1*, or *AURKAIP1* methylation levels and patient age, sex, or translocation status, suggesting that these epigenetic alterations are independent of demographic and cytogenetic factors. Overall, continuous expression analysis demonstrated that *AURKA*, *AURKB*, *AURKC*, and *E2F1* expression levels were significantly higher in AML patients, whereas *AURKAIP1* expression levels were significantly lower. Promoter methylation analysis revealed a significant association only for *AURKAIP1*, indicating a disease-associated epigenetic alteration that may support its relevance as a preliminary biomarker candidate in AML.

#### 3.2.2. Diagnostic Performance of Aurora Kinase–Related Genes

**Figure 3:**
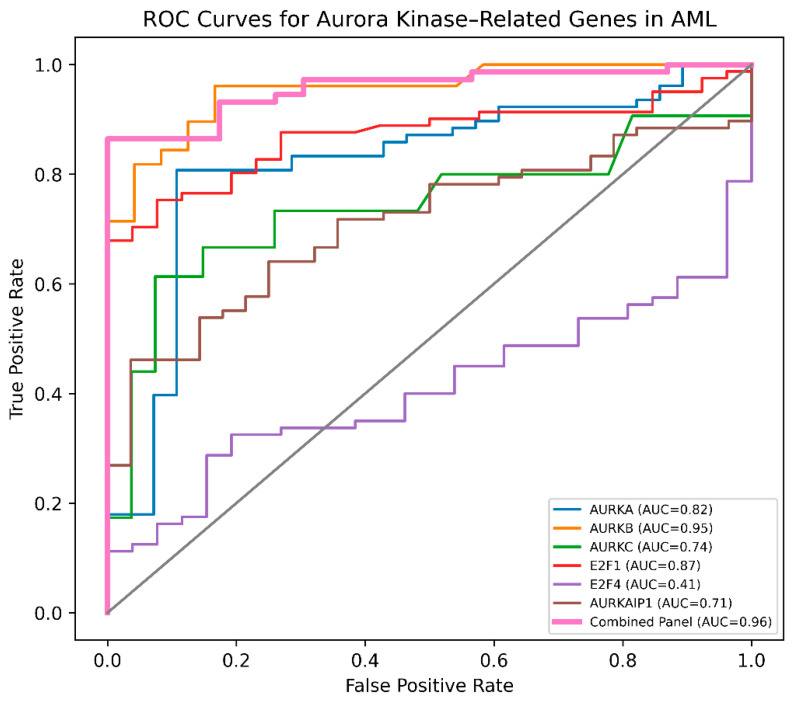
Receiver operating characteristic (ROC) curves evaluating the diagnostic performance of Aurora kinase–related genes in distinguishing AML patients from healthy controls. (*AURKB* showed the highest diagnostic accuracy (AUC = 0.95), followed by *E2F1* (AUC = 0.87) and *AURKA* (AUC = 0.82), whereas *AURKC* and *AURKAIP1* showed moderate diagnostic value and *E2F4* showed no discriminatory performance. The combined biomarker panel including *AURKA*, *AURKB*, *E2F1*, and *AURKAIP1* demonstrated improved diagnostic accuracy (AUC = 0.96) compared with individual genes.

ROC curve analysis demonstrated strong diagnostic performance of Aurora kinase–related genes in distinguishing AML patients from healthy controls (Table 4 and Figure 3). ROC analyses were performed using continuous expression values, and optimal cut-off values were determined using the Youden index. *AURKB* showed excellent diagnostic accuracy (AUC = 0.95), followed by *E2F1* (AUC = 0.87) and *AURKA* (AUC = 0.82), whereas *AURKC* and *AURKAIP1* showed moderate diagnostic value (AUC = 0.74 and 0.71, respectively). In contrast, *E2F4* showed no discriminatory performance (AUC = 0.41). The inverse ROC pattern observed for *AURKAIP1* was consistent with its reduced expression in AML. Importantly, a combined biomarker panel including *AURKA*, *AURKB*, *E2F1*, and *AURKAIP1* significantly improved diagnostic performance, achieving an AUC of 0.96 with 90.3% sensitivity and 100% specificity. These findings suggest that Aurora kinase–related pathways may represent potentially clinically relevant biomarker signatures for AML detection. However, these results should be interpreted with caution, as this was a single-center retrospective study without external validation. Therefore, these markers should be considered preliminary biomarker candidates rather than clinically actionable diagnostic tools. These findings are consistent with the known biological roles of Aurora kinases in mitotic regulation and leukemogenesis.

#### 3.2.3. Univariate Logistic Regression Analysis

Univariate logistic regression analysis based on continuous gene expression values (Table 5) demonstrated that *AURKA*, *AURKB*, *AURKC*, and *E2F1* were significantly associated with AML status (*p* < 0.01). In contrast, *E2F4* showed no significant association (*p* = 0.17). *AURKAIP1* expression was also significantly associated with AML (*p* = 0.009). These findings further support the discriminatory potential of Aurora kinase–related gene expression profiles. These results were consistent with ROC findings, further supporting the robustness of the observed associations.

## 4. Discussion

In this study, we evaluated the expression and promoter methylation profiles of Aurora kinase family members *(AURKA*, *AURKB*, and *AURKC)* and their regulatory partners *(E2F1*, *E2F4*, and *AURKAIP1*) in AML. Our findings demonstrate consistent upregulation of *AURKA*, *AURKB*, *AURKC*, and *E2F1*, accompanied by reduced *AURKAIP1* expression and gene-specific alterations in promoter methylation patterns. Together, these results support the presence of coordinated transcriptional and epigenetic dysregulation involving mitotic regulators in AML.

Mitosis is a tightly regulated and evolutionarily conserved process that ensures accurate segregation of genetic material into daughter cells [26]. Disruption of mitotic regulation is a hallmark of cancer and contributes to genomic instability, a key feature of leukemogenesis [27]. Aurora kinases (*AURKA*, *AURKB*, and *AURKC*) are serine/threonine kinases that play central roles in mitotic progression and cell-cycle regulation, and their overexpression or amplification can disrupt mitotic checkpoints, promote chromosomal instability, and drive malignant transformation [27,28]. In line with previous studies, we observed significant upregulation of *AURKA*, *AURKB*, *AURKC*, and *E2F1* in AML patients. Among these, *AURKB* emerged as the most robust marker, demonstrating the highest diagnostic performance (AUC = 0.95) and consistent upregulation, and has been widely associated with tumor aggressiveness across multiple malignancies [29,30,31,32,33]. *AURKA* also showed strong and consistent overexpression, supporting its established role in mitotic regulation and leukemogenesis [34,35]. Although *AURKC* expression was significantly increased, its biological role in AML remains less clearly defined and may be more context-dependent [36,37,38,39,40]. Similarly, *E2F1* upregulation is consistent with its role as a key transcriptional regulator of cell-cycle progression; however, its contribution appears less specific compared to Aurora kinases [41]. In contrast, *AURKAIP1* displayed reduced expression accompanied by disease-associated promoter methylation, but the lack of correlation between methylation and gene expression suggests that its role in AML may be indirect or mediated through alternative regulatory mechanisms. Taken together, these findings support a hierarchical model in which *AURKB* and *AURKA* represent the most consistent and biologically prominent markers, while *AURKC*, *E2F1*, and *AURKAIP1* may reflect complementary or context-dependent molecular alterations.

Because transcriptional and post-translational regulation play a key role in Aurora kinase expression [42], we also evaluated genes involved in Aurora kinase regulation, including *E2F1*, *E2F4*, and *AURKAIP1*. Members of the *E2F* family are key regulators of cell proliferation, differentiation, and apoptosis, and they directly control transcription of several cell-cycle genes [43]. *E2F1* acts as a transcriptional activator, particularly during the G1/S transition, and its overexpression has been reported in multiple cancers, including breast, lung, ovarian, and colorectal malignancies [44]. In agreement with these findings, *E2F1* expression was significantly elevated in AML patients and showed a positive correlation with *AURKA* expression, consistent with previous reports indicating that *E2F1* regulates *AURKA* transcription [45].

In contrast, *E2F4* primarily functions as a transcriptional repressor involved in maintaining cells in the G0/G1 phase in cooperation with pRB [46]. Although *E2F4* has been reported to act as an oncogenic factor in certain cancers [47], its role remains context-dependent. In our study, *E2F4* expression did not differ significantly between AML patients and controls, and no association was observed between *E2F4* and *AURKB* expression, which may reflect the complex regulatory functions of *E2F4* in different cellular contexts.

*AURKAIP1* is involved in the degradation of *AURKA* following mitosis, although its regulatory mechanisms remain incompletely understood [48]. Literature on *AURKAIP1* expression and methylation in cancer is limited. In our cohort, *AURKAIP1* displayed reduced expression and disease-associated promoter methylation; however, the lack of correlation between methylation, gene expression, and *AURKA* levels suggests that its functional role in AML may be indirect or context-dependent.

Epigenetic alterations are another hallmark of cancer, leading to heritable changes in gene expression without altering the DNA sequence [49]. In this study, promoter methylation of *E2F1*, *E2F4*, and *AURKAIP1* was evaluated to explore potential epigenetic regulation. Although *E2F1* showed high methylation frequency, no correlation with gene expression was observed, suggesting alternative regulatory mechanisms. Similarly, *E2F4* methylation levels were significantly lower in AML patients but were not associated with expression levels. In contrast, *AURKAIP1* promoter methylation was detected in a substantial proportion of AML patients but not in controls, indicating a potential disease-associated epigenetic event; however, its lack of correlation with gene expression suggests that its functional relevance remains unclear. Notably, methylation patterns were not associated with patient age, sex, or translocation status, supporting the interpretation that these alterations may reflect disease-related molecular changes rather than subgroup-specific effects. Nevertheless, these findings require confirmation in larger, well-stratified cohorts.

The ROC analysis suggests that Aurora kinase–related genes may have potential as preliminary biomarker candidates for AML, with *AURKB* and *E2F1* showing relatively strong diagnostic performance and improved classification when combined in a panel [50]. Accordingly, these results should be considered preliminary, and further validation in independent, multicenter, and prospective cohorts is required before clinical application. Given the minimally invasive nature of peripheral blood sampling [51], Aurora kinase–related expression signatures may nevertheless hold translational potential.

To our knowledge, this is the first study evaluating *AURKAIP1* promoter methylation in AML. While the use of MIQE-compliant methodology and the consistency of alterations across multiple genes support the robustness of the findings, few limitations should be considered. Molecular analyses were performed using PBMC fractions rather than purified leukemic blast populations and therefore reflect a heterogeneous cell population that may include both malignant and non-malignant cells. In addition, the absence of blast percentage data limited the ability to assess the contribution of leukemic burden to the observed molecular patterns. Consequently, the findings should be interpreted as peripheral blood-based molecular signals rather than blast-specific alterations. Further limitations include incomplete clinical and molecular annotation, including key prognostic markers (e.g., *ELN* risk, *FLT3*, *NPM1*, *TP53*) and survival data, which precluded comprehensive prognostic analyses. The cross-sectional design limits causal inference, and functional validation of *AURKAIP1* methylation was not performed. Moreover, although MSRE-qPCR is a practical method, it lacks single-CpG resolution compared to sequencing-based approaches.

Taken together, the consistent gene expression patterns and their alignment with previously reported AML-related pathways support the biological relevance of these findings. Peripheral blood-based molecular profiling may represent a clinically feasible and minimally invasive approach for biomarker development, particularly in settings where bone marrow sampling is limited. However, further validation in refined cellular models and independent cohorts is required to confirm the specificity and clinical utility of these alterations. Therefore, these markers should be considered preliminary rather than ready for clinical implementation.

## Figures and Tables

**Table 1:** Primers used for gene expression analysis (RT-qPCR).

Genes	Primers	Product Size (bp)
***AURK A***	F: 5′-ATT TTG GGT CAG TAC ATG CT-3′ R: 5′- GTC CAG GGT GCC ACA GAG A-3′	65 bp
***AURK B***	F: 5′-CTG CGC AGA GAG ATC GAA ATC-3′ R: 5′-AAT AGT TGT AGA GAC GCA GGA TGT TG-3′	67 bp
***AURK C***	F: 5′-GCT GCA GAA AAG CGA GAA ATT AG-3′ R: 5′-ATC TGC CAA CTC CTC TAT TAT CG-3′	64 bp
***E2F1***	F: 5′-GGA CTC TTC GGA GAA CTT TCA GAT-3′ R: 5′-GGG CAC AGG AAA ACA TCG AT-3′	69 bp
***E2F4***	F: 5′-ACC TCC TTT GAG CCC ATC AA-3′ R: 5′-CGT GTG GGA TCA AAG ATT TCT-3′	85 bp
***AURKAIP1***	F: 5′-CTG AGA CGC AAG CAG ATC AAG T-3′ R: 5′-CCT TTA GCC CCG CCT TCA-3′	67 bp
***ZNF207***	F: 5′-AAG TTG ATC CAT CCA GAT GAG GAT-3′ R: 5′-CGA GGA AGA TTA CGT TGATAC TTA GGT-3′	117 bp

**Table 2:** Primers used for methylation analysis.

Genes	Primers	Primers Size (bp)
***E2F1***	F: 5′-GGTACCATCCGGACAAAG-3′	18 bp
	R: 5′-GTTAAAGCCAATAGGAACCG-3′	20 bp
***E2F4***	F: 5′-GGAAGCGGAAGCAGTAAC-3′	18 bp
	R: 5′-GACTGCTCACCACCAAGTT-3′	19 bp
***AURKAIP1***	F: 5′-CCTTCCACGTAACTCCACTT-3′	20 bp
	R: 5′-GGATTGTGGGAAATGTAGTTT-3′	21 bp

**Table 3:** Characteristics of the patient and control groups.

	Patients	Healthy Controls
**Sex**		
**Male**	39 (47%)	15 (54%)
**Female**	44 (53%)	13 (46%)
**Age**		
**<30**	17 (20%)	1 (4%)
**30–50**	32 (39%)	16 (57%)
**>50**	34 (41%)	11 (39%)
**Subtype**		
**M1**	4 (5%)	
**M2**	2 (2%)	
**M3**	16 (19%)	
**M4**	10 (12%)	
**M4–M5**	4 (5%)	
**M5**	4 (5%)	
**M0**	43 (52%)	

**Table 4:** ROC analysis of Aurora kinase–related genes for distinguishing AML patients from healthy controls. (Optimal cut-off values were determined using the Youden index).

Gene	AUC	Sensitivity (%)	Specificity (%)	Cut-Off	95% CI
***AURKA***	0.82	80.8	89.3	0.297	0.74–0.90
***AURKB***	0.95	96.1	83.3	0.003	0.90–0.99
***AURKC***	0.74	61.3	92.6	0.005	0.64–0.84
***E2F1***	0.87	67.9	100	0.124	0.79–0.94
***E2F4***	0.41	28.8	84.6	1.477	0.29–0.53
***AURKAIP1***	0.71	46.2	96.4	0.287	0.61–0.81
**Combined Panel**	**0.96**	**90.3**	**100**	0.595	**0.94–1.00**

**Table 5:** Univariate logistic regression analysis of Aurora kinase–related genes for predicting AML status. (Odds ratios were calculated using continuous ΔCt values. Because lower ΔCt values indicate higher gene expression, odds ratios below 1 reflect an association between higher expression and AML status).

Gene	OR	95% CI	*p*-Value
***AURKA***	0.31	0.17–0.55	<0.001
***AURKB***	0.41	0.28–0.60	<0.001
***AURKC***	0.68	0.53–0.88	0.003
***E2F4***	1.25	0.91–1.72	0.17
***E2F1***	0.42	0.28–0.63	<0.001
***AURKAIP1***	1.49	1.10–2.00	0.009

## Data Availability

The data presented in this study are available from the corresponding author upon reasonable request due to ethical and privacy restrictions.

## Supplementary Materials

The following supporting information can be downloaded at https://www.mdpi.com/article/10.3390/cimb48040378/s1.
