# Supplementary material for: Dysregulation of Aurora Kinases and AURKAIP1 Promoter Methylation as Potential Peripheral Diagnostic Biomarkers in Acute Myeloid Leukemia
Source: Curr Issues Mol Biol. 2026 Apr 5;48(4):378. doi: 10.3390/cimb48040378 (PMC13115527; doi:10.3390/cimb48040378)
Supplement: Supplementary file 1 [file cimb-48-00378-s001.zip › cimb-4208560-supplementary.pdf]

Supplementary Table S1.FDR-adjusted p-values(Benjamin-Hochberg)

Gene	Raw p-value	FDR-adjusted p-value
**AURKA**	<0.001	0.0012
**AURKB**	<0.001	0.0012
**AURKC**	<0.001	0.0012
**E2F1**	<0.001	0.0012
**AURKAIP1**	0.001	0.0012
**E2F4**	0.16	0.16
